# Polysaccharide-Based In Situ Self-Healing Hydrogels for Tissue Engineering Applications

**DOI:** 10.3390/polym12102261

**Published:** 2020-10-01

**Authors:** Sheila Maiz-Fernández, Leyre Pérez-Álvarez, Leire Ruiz-Rubio, Jose Luis Vilas-Vilela, Senentxu Lanceros-Mendez

**Affiliations:** 1 BCMaterials, Basque Center for Materials, Applications and Nanostructures, UPV/EHU Science Park, 48940 Leioa, Spain; sheila.maiz@bcmaterials.net (S.M.-F.); leire.ruiz@ehu.es (L.R.-R.); joseluis.vilas@ehu.es (J.L.V.-V.); senentxu.lanceros@bcmaterials.net (S.L.-M.); 2 Macromolecular Chemistry Group (LABQUIMAC), Department of Physical Chemistry, Faculty of Science and Technology, University of the Basque Country, UPV/EHU, Barrio Sarriena, s/n, 48940 Leioa, Spain; 3 IKERBASQUE, Basque Foundation for Science, 48013 Bilbao, Spain

**Keywords:** polysaccharide, self-healing, in situ hydrogels, dynamic bonds, injectability, tissue engineering

## Abstract

In situ hydrogels have attracted increasing interest in recent years due to the need to develop effective and practical implantable platforms. Traditional hydrogels require surgical interventions to be implanted and are far from providing personalized medicine applications. However, in situ hydrogels offer a wide variety of advantages, such as a non-invasive nature due to their localized action or the ability to perfectly adapt to the place to be replaced regardless the size, shape or irregularities. In recent years, research has particularly focused on in situ hydrogels based on natural polysaccharides due to their promising properties such as biocompatibility, biodegradability and their ability to self-repair. This last property inspired in nature gives them the possibility of maintaining their integrity even after damage, owing to specific physical interactions or dynamic covalent bonds that provide reversible linkages. In this review, the different self-healing mechanisms, as well as the latest research on in situ self-healing hydrogels, is presented, together with the potential applications of these materials in tissue regeneration.

## 1. Introduction

Hydrogels are three-dimensional networks [1,2,3] able to absorb large amounts of water or biological fluids keeping their form and without dissolving. The use of these materials in the biomedical field dates back to 1960 when Wichterle and Lim [4] developed the first synthetic hydrogel based on hydroxyethyl methacrylate (HEMA) and ethylene glycol dimethacrylate (EGDMA), as a crosslinking agent, which was used for the production of contact lenses. Owing to their valuable properties, such as biocompatibility [5,6,7], elasticity [5,8] and high water content, these materials have been explored for a large number of potential applications, in particular in the biomedical area. Indeed, they offer good opportunities for the design of scaffolds for tissue engineering applications [9,10,11,12] as well as for the controlled delivery of bioactive molecules (proteins, drugs or growth factors, among others) [12,13,14,15,16]. However, synthetic hydrogels are known to be mechanically weak, which usually represents a limitation for certain bioapplications, which require high tensile strength and toughness, as is the case for tissue engineering. Besides, the use of a pre-formed hydrogel as an implant in the desired site of the body causes the need for a surgical intervention that is often undesirable and can lead to adverse side effects. With the aim of overcoming these disadvantages, the interest in the manufacture of in situ hydrogels has grown over the years [2]. In situ hydrogels or injectable gels are a type of material capable of responding to an external stimulus, such as temperature, pH or light, causing a sol-gel transition or chemical reaction that leads to new covalent bonds. That is, before being injected into the damaged place of the body they appear in liquid form and, once within the physiological medium and thanks to the variation in an external stimulus or to a covalent bonding formation, among others, they gel quickly [17,18,19,20]. The application method of these materials is non-invasive due to their localized action and also allows one to avoid the design of a customized scaffold for each patient because the hydrogels adjust perfectly to the shape, size and irregularities of the area to be repaired. In situ forming hydrogels can be divided into two main groups: physically crosslinked hydrogels, which are the ones formed by reversible physics interactions, such as hydrogen bonds, electrostatic or hydrophobic interactions [21,22,23], and chemically crosslinked hydrogels, which are based on chemical reactions such as Schiff-base, Diels-Alder, Thiol-ene or Michael reactions which lead to new permanent covalent bonds. According to the hydrogel nature, they can be or cannot be degraded under physiological conditions, and consequently they can be stable or permanent. 

The use of synthetic polymers has been pushed into the background due to the growth in the use of polysaccharides [6,15,24,25]. Polysaccharides are abundant and available from renewable sources such as algal [26,27], crustaceans [12,28] or plants. Thus, they present a wide variety of compositions and properties that cannot be easily mimicked in the lab and the ease of their production makes them more valuable and cheaper than synthetic polymers. Although synthetic polymers usually offer the possibility to develop more robust hydrogels, with more controlled properties (molecular weight, functional reactive groups, or density) and many of them can offer good biocompatibility, they are not commonly biodegradable, unlike polysaccharides. Moreover, polysaccharides have been extensively and successfully used in a wide variety of applications in the biomedical field thanks to their similarity to natural cell tissues (extracellular matrix, ECM). Due to this, the use of polysaccharides is preferred over synthetic polymers, and their hydrogels are one of the most promising materials within the scope of advanced biomaterials [29,30,31,32]. Nevertheless, in order to enhance their mechanical properties they are usually combined with synthetic polymers [3] or even nanoparticles leading to hybrid systems [10]. Alginate [33,34,35], chitosan [36,37], hyaluronic acid [38] and cellulose are among the most used polymers in this field for applications that include gene delivery [39,40,41], wound healing [42,43,44] or controlled delivery of encapsulated bioactive molecules [45,46].

Recently, hydrogel investigations have focused on in situ hydrogels with the self-healing property able to autonomously heal damage. These hydrogels offer the possibility to maintain the integrity of the hydrogel and preserve its mechanical stability leading to a material with a longer useful life due to hydrogels being able to recover to their original shape after their disintegration without the need of any external interventions, which is also an added value for traditional in situ hydrogels [47,48,49]. Self-healing property is not a new concept in the scope of living tissue. Certainly, human skin is commonly known to regenerate itself to its initial shape and functionality after being damaged. Until today, numerous in situ hydrogel formulations with self-healing properties have been developed for different applications, in particular in the biomedical field [50]. Thus, in situ self-healing is an essential feature for those materials to be used in applications on which the structure can be damaged by movement or can degrade before completing their goal. Therefore, self-healing property confers to the material’s important advantages, such as increase in safety, cost reduction due to the lack of the need of replacements and increase in useful life. Thus, the development of polysaccharide-based hydrogels with the capacity to act against damage and self-repair represents nowadays a promising new generation of materials able to substitute traditional hydrogels in the near future.

Despite many works having been reported on the study of hydrogels with self-healing properties over the years [51], only a few works include the study of the in situ forming mechanisms of these self-healing materials. In this review, we summarize recent advances in self-healing and in situ forming polysaccharide hydrogels and their more recent applications.

## 2. Self-Healing Mechanisms

Hydrogels with autonomous self-healing abilities must be designed according to specific and strict criteria to allow the repairing of the damage autonomously, in the desired scale of time, and maintaining in a repeatable and efficient way the rheological and mechanical properties, among others. This design must also take into account the inexpensive and nontoxic nature of the employed materials and procedures, which makes polysaccharides interesting candidates as bioresource and biodegradable polymers. However, the self-healing capability of these polymers is restricted to their suitability to be involved in a dynamic equilibrium of interactions, in which reversible dissociation and recombination of certain units take place through the matrix along the time. That is, they must be provided with functional groups that promote reversible and strong interactions responsible for the self-repairing action. Thus, self-healing mechanisms require certain functionalities acting as ‘‘mobile phases” to fill and provide linkages between the damaged parts of the gels [52]. Certainly, the self-healing ability is based on the capability of carrying out the dissipation of mechanical energy during the rupture, and this can be obtained by mean of sacrificial bonds. These specific bonds are disrupted, avoiding the cleavage of the polymeric main backbone, and they are continuously re-established for the self-reparation of the network.

**Figure 1:**
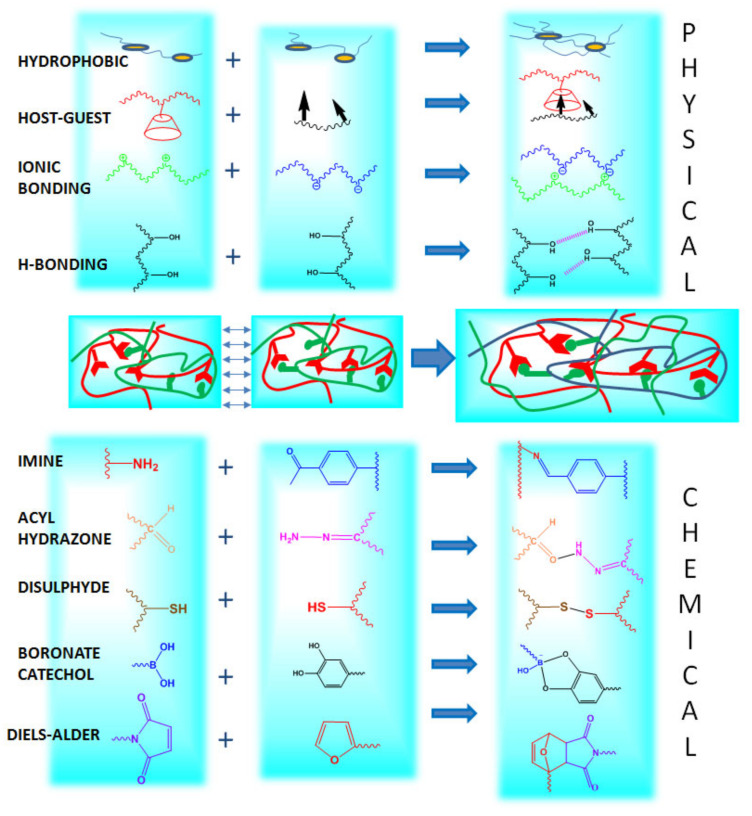
Illustrative scheme of the different interactions/reactions that promote the self-healing process.

Typically, the mechanisms promoting self-healing capability, which are based on dynamic interactions, can be classified as covalent reactions that lead to the chemical crosslinking of the polymer/s, and supramolecular assembly, based on physical interactions (Figure 1).

Generally, dynamic covalent equilibriums are processes slower than those of the supramolecular chemistry, and switching usually requires an external stimulus or, sometimes, even the presence of a catalyst is demanded for applicable time frameworks. Nevertheless, this disadvantage can be interestingly exploited in order to develop stimuli-responsive materials with new and tunable properties. Further, dynamic covalent linkages promote more stable self-healed networks than fast but fragile supramolecular bonds [53]. Nevertheless, the reversible nature of the dynamic linkages, both physical and chemical, implies a weaker character and subsequently, poorer mechanical properties than fully permanent covalent networks [48]. Different approaches have been investigated to overcome this issue and to obtain robust and highly stretchable self-healing hydrogels, such as incorporating fillers leading to composite materials, or adding a second dynamic or non-reversible network, leading to interpenetrating polymer networks (IPNs). As expected, these approaches lead to a restriction of the self-repairing ability and full recovery is not reached as a consequence of the cleavage of the irreversible bonds.

Dynamic covalent bonds mainly include phenylboronic ester bonds (boronate ester), dynamic imine bonds (dynamic Schiff base), disulfide bonds, acyl hydrazones bonds and Diels-Alder reactions [48]. On the other hand, main dynamic non-covalent interactions correspond to hydrogen bonding, hydrophobic interaction, host-guest interaction and ionic interaction [48]. The combination of the indicated dynamic forces has been also explored to achieve self-healing networks governed by multiple intermolecular interactions.

### 2.1. Dynamic Covalent Bonding

Dynamic covalent bonding is one of the main approaches explored for the development of self-healing hydrogels because it combines the stability of the traditional permanent chemical bonds with the reversibility of the physical interactions. As indicated before, this reversibility relies on the fact that bonds can be disrupted and reformed autonomously through all the hydrogels due to an equilibrium condition. This is, according to the external conditions, they can act as reversible physical bonds or can behave as permanent covalent linkages [54].

Furthermore, dynamic covalent bonding inherently provides hydrogels with the unique property of being able at the same time to respond to external stimuli such as pH, temperature or redox species [52].

#### 2.1.1. Imine Bonds

Imines, also referred to as Schiff bases, present a C=N bond as a result of the nucleophilic attack of amine primary moiety to aldehyde or ketone groups. Interestingly, highly crosslinked networks can be prepared by the condensation between polyamines and dialdehydes. Both aliphatic and aromatic dialdehydes have been used in the preparation of self-healing hydrogels, showing that aromaticity promotes stability and enhances the final mechanical properties of the gels [55]. Indeed, the benzaldehyde-difunctionalized poly(ethylene glycol) has been one of the most exploited dialdehyde to form self-healing hydrogels through imines formation by simple mixing with polymers functionalized with amino groups, typically chitosan and its derivatives [56].

There are not many different aldehydes, apart from benzaldehyde-difunctionalized poly(ethylene glycol), that have been used to form Schiff-base-mediated self-healing hydrogels. For instance, a multifunctionalized aldehyde based on chondroitin sulfate has been expressly functionalized and also explored for the preparation of polymeric networks crosslinked by dynamic imines [57]. This strategy has been also applied to hyaluronic acid in order to obtain the aldehyde-modified polysaccharide with robust self-healing behavior after reaction with cysteine diamine [58].

Interestingly, Schiff bases are usually sensitive to hydrolysis under acidic conditions [54] and as a result, these types of hydrogels present pH-responsive formation as an adequate external pH is required as a condition for the condensation reaction [59].

#### 2.1.2. Acylhydrazone Bonds

Acylhydrazone bonds are dynamic covalent bonds produced by the condensation reaction of aldehydes and hydrazines. This reaction leads to more stable networks than imine-mediated crosslinking [50]. Bis(acylhydrazine)- functionalized poly(ethylene oxide) (PEO) is the typical polymer reported for the preparation of hydrogels by acylhydrazone bonds after reaction with multifunctional aldehydes such as tris[(4-formylphenoxy)methyl]ethane [60]. Further, PEO has been also employed to incorporate dialdehyde functionality, while acylhydrazine groups were present as a copolymer of *N*-isopropylacrylamide leading also by the same reaction to self-healable hydrogels [61].

As is known, mild acidic conditions are required for acylhydrazone bond formations and an acid catalyst is usually needed for the reaction because they are not easily formed at neutral conditions. This implies that acylhydrazone crosslinking can be modulated by changing the external pH and, consequently, the healing process only takes place in a restricted range of pH (4 < pH < 6) [62]. This fact also becomes an advantage because sol-gel transformations take place as a response to changes of the external pH, enabling in situ gelation and easing hydrogel injectability [50].

#### 2.1.3. Disulfide Bonds

Disulfide bonds are formed by the oxidation of thiols at favorable conditions, such as neutral or alkaline pHs and room temperature. This thiol/disulfide dynamic exchange reaction represents a feasible mechanism for the synthesis of self-repairing networks. In addition, disulfide bonds represent a versatile alternative in the design of self-healing hydrogels as the crosslinking reaction can be initiated by a considerable variety of stimuli such as heat, light and external radicals [53]. Regarding the introduction of external radicals, it is worth highlighting that this leads to the disruption of disulfide bonds producing sulfur radicals that compete in chain transfer, limiting the thiol-disulphide exchange and thus the self-healing ability [54]. In this sense, high concentrations of external radicals, due to an excess of radical initiator in feed or a posterior long exposure to air, lead not only to the restriction of the self-mending property but to the final degradation of the network [52].

This characteristic can be readapted for preparing photodegradable and photoadaptable materials. This is the case of the dynamic covalent hydrogels formed by the oxidation of multi-functionalized thiol-poly(ethylene glycol) leading to disulfide crosslinked networks. These networks were swollen in the presence of a water soluble photoinitiator that generates, after exposure to the appropriate wavelength (365 nm), radicals that promote the cleavage of the disulfides and, finally, the degradation of the network when high concentrations of radicals are achieved [63].

#### 2.1.4. Boronate-Ester Complexation

Reversible covalent bonds named boronate ester bonds are formed when diols are complexed with boronic acid and its derivatives, such as phenylboronic acid or polymers functionalized with phenylboronic acid. With respect to boronic acid functionality, it is usually incorporated in polymeric backbones, by the polymerization of a boronic acid-containing monomer in the presence of a radical crosslinker, or by grafting on pre-synthesized polymeric networks [64]. Regarding diol functionality and inspired by mussel adhesive proteins, catechol group is one of the most employed diols in the generation of boronate ester complexes that lead to self-repairing networks. Typically, branched catechol-derivatized poly(ethylene glycol) (PEG) is employed for the complexation with 1,3-benzenediboronic acid (BDBA) in order to prepare self-healing hydrogels. Nevertheless, oxidation of the catechol groups is an associate problem to overcome because it restricts the self-healing ability of the hydrogels [52].

The boronate ester complexation is strongly affected by the pH which limits the application of boronate-catechol complexation to biological applications [50]. According to the pKa of the employed boronic acid, it has been demonstrated that an adequate self-healing ability exists at pH near to the pKa of the used phenylboronic acid, while high rigidity prevents self-repairing at pH higher than the pKa, and even sol formation is observed at pH values below the pKa [65]. Regarding the diol, pH values higher than the pKa of diol allow its ionization and consequent boronate-ester reaction [66]. In addition, it has been shown that the robustness of the self-healing property is influenced by the exact conformation of the diol along the polymeric chains [67].

The binding ability of phenylboronic acid to diols extends to glucose, and consequently glucose-responsive assemblies are obtained within boronic acid-based hydrogels. Therefore, these gels display promising applications in drug delivery due to their unique glucose-dependent swelling behavior [68].

#### 2.1.5. Diels-Alder “Click Chemistry”

The Diels-Alder reaction is a [4 + 2] cycloaddition between a conjugated diene and a substituted alkene, also called the dienophile, generating a cyclohexene derivative. These dienophiles must have electron-poor character as a consequence of the presence of electron-withdrawing substituents, to enable the reaction with the diene. Diels-Alder reaction is a high selective reaction [52] that presents “click” properties and offers large possibilities to the preparation of polymeric networks from multifunctional diene and dienophile compounds [53].

The self-recovering properties of thus obtained networks are based on the thermal reversibility of Diels-Alder reactions, because the formed compounds are not stable at high temperatures. In these conditions (100–150 °C), the reverse reaction can take place, regenerating the diene and the dienophile. Accordingly, the use of the Diels-Alder reaction as a mechanism for the preparation of self-repairing hydrogels applied to the biomedical field is restricted due to the required high temperatures and long times needed for the disruption and reforming of the bonds for self-healing processes [69]. For this reason, Diels-Alder chemistry has been employed preferably combined with other dynamic bonds, both chemical [34] and physical [34], in order to prepare hydrogels with efficient self-healing ability.

The maleimide group presents a relatively high reactivity as dienophile for Diels-Alder reactions, and hydrogels formed by reaction between maleimide groups and furan attached to polymeric chains are the typical examples of thermally reversible crosslinked networks by Diels-Alder chemistry [69]. However, the restricted mobility derived from the steric hindrance seems to limit the self-healing process. For this reason, multi-functional small molecules are preferred as monomers to develop crosslinked networks, and they have shown successful self-repairing action. Nevertheless, extended reaction times and the high temperature requirement continue to limit the application of these hydrogels. In this respect, reactivity has been increased at room temperature by the use of 1,2,4-triazoline-3,5-dione as dienophile unless reversibility is compromised, which has been overcome with the use of indole as diene [69].

### 2.2. Physical Bonding

#### 2.2.1. Hydrogen Bonding

When a hydrogen atom is bonded directly to a highly electronegative atom (O, N, or F) electrostatic interactions lead to reversible interactions called hydrogen bonds. Thus, polymer chains provided by hydrogen donor groups (electropositive atoms) and hydrogen acceptor groups (electronegative atoms) result in H-bonding and the formation of reversible crosslinked polymeric networks. Despite this type of interaction being relatively weaker than dynamic covalent bonds or ionic bonds, it is strong enough to provide gelation and self-healing properties [52]. In fact, hydrogen bonding is one of the most common approaches to fabricate self-healing hydrogels by physical interactions. For instance, poly(vinyl alcohol) is an hydrophilic polymer that possesses hydroxyl groups able to establish H-bonding and generate networks with self-healing ability when it is present in a sufficient concentration. However, the unspecific nature of this interaction hinders the recombination after extended fragmentation periods due to the loss of free surface hydroxyl groups by inter- or intra- H-bonds [70]. As a consequence of this lack of specificity, there are considerable variables that compromise the self-repairing capacity, such as the extent of the protonation or the steric hindrance of the groups [50]. This dependence of the ability to recombine H-bonding with the external conditions enables that they can be exploited to prepare pH-mediated self-healing hydrogels [71].

Catechol, or 2-hydroxyphenol, groups, which, according to their chemical structure, are also able to form H-bonding, have revealed to have an active role in accelerating the H-bonding mediated self-healing process. Thus, grafting with catechol groups has emerged as an alternative to developing self-healing hydrogels by means of H-bonding interactions [72].

#### 2.2.2. Ionic Bonding

Electrostatic attractive interactions between oppositely charged ions and polyelectrolytes represent the ionic mechanisms for self-healing properties. These interactions produce the crosslinking of a polyelectrolyte in the presence of an oppositely charged ion or polymer by simple mixture in solution. This straightforward mechanism, however, is also highly dependent on the external conditions, such as the net ionization degree of the weak polyelectrolytes, which differ according to the pH or the presence of salts [48]. In addition, these electrostatic networks have shown dependence with the migration ability of both the free ions (migration velocity) and the free polyelectrolyte (for example, influenced by the molecular weight).

Mixing polyanions, like poly (acrylic acid), with ferric ions has been widely used as a strategy to prepare networks with dynamic ionic bonding; these networks have shown the ability to be restored after damage while presenting acceptable mechanical properties [73]. 

Catechol moiety shows coordinative bonding with cations like ferric or cupric ions. According to this, multi-catechol polymeric derivatives have shown coordinative crosslinking with the cited ions when they are included in high concentrations, leading to the formation of hydrogels with self-healing ability [74].

#### 2.2.3. Host-Guest Interaction

Host-guest interactions consist of the specific assembly between two (or more) compounds by inclusion thanks to their complementary structures. Thus, a species named “the guest” is included within another molecule known as “the host” by non-covalent interactions, such as hydrogen bonds or electrostatic interactions. The reversible nature of the interactions that take part in this assembly confers a dynamic character to the crosslinking formed by this way, which has been employed in the self-healing processes. Indeed, host-guest interactions are among the most explored supramolecular interactions in the synthesis of crosslinked networks with self-healing capability. The robustness of the crosslinking depends on the strength of the host-guest interaction that differs according to the affinity between the selected host and guest species [75].

Host molecules are macrocycles characterized by including an internal cavity that allows the accommodation of different guest molecules. In the last decades, different host cycles have been studied for the synthesis of hydrogels by inclusion complexation, such as cyclodextrins (CDs) or cucurbit[n]urils (CB[n]) or calixarenes [76]. Among all of them, cyclodextrins (CD) are undoubtedly the most known host compounds, which have been widely employed to develop self-healing hydrogels due to their biocompatibility and non-toxicity. CDs are hydrophilic molecules whose structure is represented as a truncated cone with a hydrophilic outer surface and a hydrophobic inner cavity that confers them an amphiphilic nature. CDs form inclusion complexes with small molecules and with specific parts of macromolecules. In addition, they can be easily modified chemically by means of the hydroxyl groups present in their outer surface. 

Adamantine, ferrocene, cholic acid, or azobenzene are some of the most representative guest molecules able to form a stable inclusion complex with cyclodextrins and their derivatives [76]. Therefore, functionalization of synthetic and natural polymers with both cited pendant host and guest functionalities to their complementary interaction has been the most reported via the design of host-guest mediated self-healing hydrogels. Two cases that illustrate this approach are the hydrogels formed by the association of cyclodextrin-functionalized acrylamide and ferrocene-functionalized poly(acrylic acid) [77], or that of the assembly of PEG polymer modified with cholic acid dimers with copolymers of *N*,*N*′-dimethylacrylamide grafted with CDs [78].

The case of azobenzene as guest molecule for CDs is particularly noticeable because, on the basis of the trans-cis isomerization that occurs under light irradiation, the obtained hydrogels also present a photo-responsive behavior that has been exploited to modulate the self-healing properties by photoirradiation [79]. 

Regarding hydrogels formed by inclusion in host molecules different to CDs, it is worth highlighting those formed by complexation of CB[n] macrocyclic oligomers with a few guest moieties. As example, multifunctional polymeric derivatives of viologen or naphthoxy have been typically employed with CB [8,80]. Some host-guest hydrogels like those obtained by complexation of calix [4] arene derivatives of tetra-proline with the amino acids arginine, histidine and lysine were also prepared with thermoreversible properties [81].

#### 2.2.4. Hydrophobic Bonding

Hydrophobic interactions are responsible for the aggregation of hydrophobic domains in aqueous media because of the high interfacial tension. When hydrophobic monomers are added together with hydrophilic ones into a network, hydrophobic aggregates are easily formed and reformed after being disturbed. Thus, micelles formed after surfactant addition or liposomes can be employed as crosslinking points to form polymeric networks when both hydrophilic and hydrophobic monomers are copolymerized. The hydrophobic forces can act in these networks as reforming interactions in a damaged area if hydrophobic content is adequate in the mixture.

The most followed method to the preparation of self-healing hydrogels by hydrophobic bonding is the so-called “micellar polymerization”. This methodology is based on the solubilization of hydrophobic monomers in a solution containing surfactant forming micelles and an hydrophilic monomer that copolymerizes simultaneously typically by free-radical addition [82].

One simple example is that which corresponds to the copolymerization of stearyl methacrylate with acrylamide in sodium dodecyl sulfate aqueous solution that leads to hydrogels with excellent mechanical properties and successful and fast self-healing healing behavior [83].

Factors that vary the solubilization of the hydrophobes, such as the addition of electrolytes, hinder the micellar growth, and consequently the self-healing process [82].

## 3. In Situ Self-Healing Polysaccharide-Based Hydrogels

In the following sections, the main works on polysaccharide-based in situ self-healing hydrogels are summarized. The classification is made according to the interactions or bonds created after their response to external stimuli that lead to the sol-gel transition. Based on this, different types of in situ hydrogels are shown, such as hydrophobic interaction-based hydrogels, ionically crosslinked hydrogels, hydrogels based on in situ polymerization and dynamic covalent bond-based hydrogels, among others.

### 3.1. Hydrophobic Self-Healing Hydrogels

One of the main types of hydrophobic interaction-based self-healing hydrogels is the type that responds to an external temperature change. In this field, there are numerous polymers capable of undergoing a sol-gel transition in response to temperature changes [84,85]. This transition is induced at a temperature named upper or lower critical solution temperature; UCST and LCST, respectively. In polymers, this thermosensible sol-gel transition is due to a variation in the solubility of the polymer owing to a variation between the interactions of hydrophilic and hydrophobic moieties of the polymer with water. The most interesting situation for biomedical applications is that of the LCST cases, in which the interactions between macromolecule chains and the solvent by H-bonding predominate over hydrophobic interactions at temperatures below LCST. However, when the temperature exceeds LCST, the hydrophilic interactions (polymer hydrophilic moieties-water) are weakened and the hydrophobic interactions lead to precipitated hydrogel. This behavior is known as the hydrophobic effect and offers the possibility of developing a reversible in situ matrix, which can return to solution by changing temperature. This thermal response is a promising feature because no other requirement for chemical or environmental treatment is needed, as temperature is the only external factor that takes part in the gelation process.

The main thermosensible polymers are not polysaccharides. They are poly (*N*-isopropylacrylamide) (PNIPAAm) [3,20,86] and poly(ethylene glycol)-poly(propylene oxide)-poly(ethylene glycol) block copolymer (PEO-PPO-PEO), also known as Pluronic F-127^®^ [87,88], which shows an LCST close to body temperature. Different studies have been reported with these two thermosensible polymers but, since they are not biodegradable, they typically appear in combination with polysaccharides, such as chitosan [89,90], alginate [35,91,92], cellulose [93,94] or hyaluronic acid [95,96] in order to obtain a hydrogel matrix with tunable degradation behavior. However, these materials lack the ability to self-repair because gelation is based on hydrophobic interactions that result in hydrogel precipitation.

For example, taking advantage of the thermosensitivity of a synthetic polymer such as Pluronic^®^ (LCST ≈ physiological temperature), a thermosensitive structure capable of self-repairing by the host-guest mechanism was obtained by host-guest interaction with alginate derived with β-cyclodextrin (β-CD) [97]. This double-crosslinked network was obtained due to the interaction between host polymer (Pluronic ^®^) and guest moieties of alginate-β-CD. In addition, at a temperature around physiological temperature and thanks to the rapid response of Pluronic^®^ to temperature variation; the second crosslinking was achieved as a consequence of the hydrophobic interactions between polymer chains. Moreover, the mechanical measurements reveal that the shear storage moduli of the hydrogel (30 kPa) at body temperature was maintained even after breaking the hydrogel which demonstrates their potential to be used as scaffold for biomedical applications.

Chitosan has also been successfully employed in the development of in situ hydrogels with the ability to self-repair. This is the case of hydrophobically functionalized chitosan (adding C12 groups) mixed with dodecyltrimethylammonium bromide (DTAB) and a thermosensitive vesicle (5-methyl syalic acid) [98]. The obtained gel shows good stability at temperatures around 20 °C. However, the increase in temperature favors the gel-sol transition, reverting the gel to its original form (liquid). The sol-gel transition, as in the aforementioned case, is directly related to hydrophobic interactions between the hydrophobically modified chitosan and the vesicles, which in turn play as multiple crosslinking points, leading to supramolecular hydrogels. The self-repairing of these gels has been thoroughly studied by rheology, where a quick repair (almost 10 s) was obtained, which was kept for four cycles. Therefore, thermosensitive vesicles offer the possibility of forming reversible dynamic bonds thanks to the hydrophobic interactions and can be embedded or de-embedded from the vesicle bilayer.

### 3.2. Ionically Induced Self-Healing Hydrogels

Ionically crosslinked hydrogels are obtained from a mixture of two or more polyelectrolytes in a suitable pH which leads to the protonation or deprotonation of the ionizable moieties which are through the structure. Typically, natural polymers with pendant ionizable groups, such as alginate [33,34,35], chitosan [36,37], hyaluronic acid [38] and cellulose have been extensively used in the development of ionically crosslinked systems and ionically induced self-healed hydrogels. As there are several pH changes in physiological medium, such as upper stomach (4.0–6.5), lower stomach (1.5–4.0) or saliva (6.5–7.5), hydrogels that are able to be crosslinked due to pH changes are promising materials for biomedical applications, e.g., as sensors and actuators, as scaffolds for mimicking different microenvironments for 3D culture or as bioactive molecular carriers [99,100].

The most typical polysaccharide within ionically crosslinked self-healing hydrogels is alginate. Alginate is an anionic polysaccharide, thanks to the carboxylic groups that are repeated throughout its structure. This biopolymer shows an ability to create gels when cations, such as calcium (Ca^2+^), zinc (Zn^2+^) or magnesium (Mg^2+^) are added [33,34,35]. Those cations, at a specific pH ≈ 4.5 (pKa_alginate_ = 3.5) interact with the carboxylate groups of the mannuronic acid and guluronic acid units and an ionic hydrogel with self-healing by dynamic and reversible ionic bonds is formed. The alginate/CaCl_2_ system has been used for several applications in biomedicine. However, the main drawback of these materials under physiological conditions is their poor mechanical stability. For this reason, chemical crosslinking is normally added to these hydrogels by modifying the biopolymer with acrylic or vinyl groups, among others, to improve their mechanical properties [101]. Often, these double crosslinking systems do not present total degradation or an efficient ability to self-repair. Consequently, interest in combining oppositely charged polysaccharides to obtain biodegradable and biocompatible matrices with self-healing properties by ionic bonds has grown over the last years.

**Figure 2:**
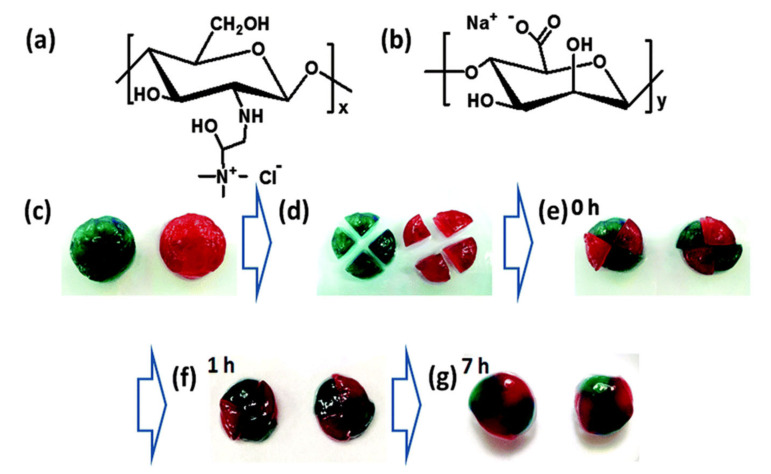
Self-healing process of hydroxypropyltrimethylammonium chitosan chloride (HACC)-alginate-based polycomplex. (**a**,**b**) Chemical structure of HACC and alginate respectively. (**c**–**e**) Two coloured disk-shaped hydrogels cut into 4 pieces and put back together. (**f**,**g**) Self-healed hydrogel after 1 and 7 h. Reproduced from Y. Ren et al. A self-healing hydrogel formation strategy via exploiting endothermic interactions between polyelectrolytes, 2016 [102], with permission from The Royal Society of Chemistry.

This is the case of Ren et al. [102] who developed a reversible in situ hydrogel with self-healing ability based on the oppositely charged polysaccharides, alginate and chitosan. However, the number of interactions between both polysaccharides is extremely high and the obtained hydrogels resulted so strong, that the self-healing process was hindered. Therefore, chitosan was substituted by 2-hydroxypropyltrimethylammonium chitosan chloride (HACC), because the large size of the quaternary ammonium cationic groups leads to an increase in the distance with alginate and, as a result, the ionic interactions became weaker, favoring the self-healing process which takes place in 7 h without any external stimuli (Figure 2). Isothermal titration calorimetry (ITC) reveals that the electrostatic interactions between alginate and HACC were endothermic, this is, the hydrogel formation is entropically driven due to the formation of positive peaks. The polyelectrolyte-based hydrogel exhibits promising properties such as muco-adhesion, shear-thinning and cell compatibility which would be useful for bioapplications.

**Figure 3:**
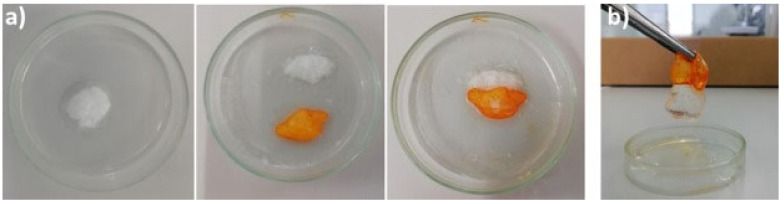
Self-healing process of chitosan and hyaluronic-acid-based polycomplex, (**a**) cut and repairing process of the system, and (**b**) repaired hydrogel. Reprinted with permission from N. Barroso et al. Self-healable hyaluronic acid/chitosan polyelectrolyte complex hydrogels and multilayers [51]. Copyright © (2020), with permission from Elsevier.

A few works have focused on hyaluronic acid and chitosan-based polycomplexes, which provide the possibility of in situ gel formation as well as rapid self-healing process thanks to ionic interactions. In this case, the mixture of chitosan and hyaluronic acid in adequate pH (~4) leads to the formation of a hydrogel with ability to self-repair in 2 min (Figure 3) [51]. In order to measure the mechanical response of the synthesized hydrogels, different cut-recovery cycles (2) were applied and the results showed that after the self-healing process a new material can be obtain with properties nearly as good as the original hydrogel, which opens a new research line with promising opportunities in the biological field.

**Figure 4:**
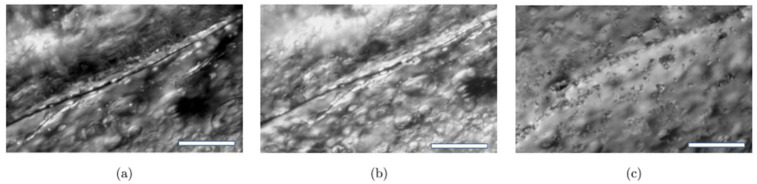
Self-healing process by microscope pictures. (**a**) Hydrogel was cut on the surface, the hydrogel surface appearance after (**b**) 15 min, (**c**) 80 min. Reproduced from S. Biswas et al. A Stimuli-Responsive Supramolecular Hydrogel for Controlled Release of Drug [103].

Another study that has revealed the interest in combining oppositely charged polysaccharides [103] used chitosan, guanidine hydrochloride and poly (acrylic acid) in order to obtain a ionically crosslinked matrix with pH-responsive ability and self-healing. When the pH of the system increases (pH ~ 8), the carboxylic acid moieties of poly (acrylic acid), the hydroxyl groups of guanidine hydrochloride and amine groups of chitosan became deprotonated and the ionic interactions between the three compounds are switched on. The self-healing study revealed that the hydrogel was able to self-heal itself in a few hours by recovering its initial shape owing to ionic interactions (Figure 4). Moreover, the gelation of the hydrogel seems to be suitable for drug delivery or 3D cell culture applications as the pH induced a rapid sol-gel transition.

Double physical crosslinking has been also exploited for the development of in situ hydrogels with self-healing properties with improved consistency. For instance, chitosan, a copolymer of acrylamide and acrylic acid, was mixed in the presence of iron (III) salt. The authors [90] worked at an specific (slightly acidic) pH where the primary amine groups of the chitosan were protonated (-NH_3_^+^) and, at the same time, the -COOH groups of the acrylic acid and the -CONH_2_ of the polyacrylamide were also in an ionized state (-COO^−^ and -CONH_3_^+^, respectively). The results of the gelation process analyzed by FTIR revealed that the trivalent cation of iron (Fe^3+^) interacts ionically with the anion formed by the deprotonation of the carboxylic groups. In turn, the free amino groups of chitosan and the -COONH_3_^+^ group of the copolymer also interacted with -COO^−^. Furthermore, as the ionization is not total, the amino and amide groups as well as the carboxylic group are capable of establishing hydrogen bonds, leading to a double-crosslinked network. Besides, synthesized hydrogels were able to achieve good self-healing capacity due to the reversible ionic and hydrogen bonds. The results of the tensile stress before and after the self-repair process demonstrated that the hydrogel maintains almost totally the mechanical stability.

Other types of ionically induced hydrogels are those based on metal-ligand interactions. This class of smart materials are composed of a ligand (electron donor, catechol for instance) and a metal ion that results in a hydrogel formed by self-assembly in a supramolecular network. Metal-ligand bonds are reversible in nature and have become the point of interest of many researchers due to the ability to rapid self-repair. Since chitosan offers suitable possibility to obtain hydrogels with excellent biocompatibility and versatility to be functionalized, its derived-based hydrogels have attached huge attention in the field of self-repair materials. Chitosan-catechol, which was formed by the modification of the polysaccharide with dihydrobenzaldehyde, was able to self-assemble by establishing coordinative interactions between catechol moiety and iron (III). These in situ hydrogels that formed in no more than 5 min also have an excellent ability to self-heal thanks to the reversible coordination bonds, which gives them the possibility of being injected subcutaneously. The results obtained from these gels demonstrate that the self-healing process occurs even after loading drugs, such as doxorubicin hydrochloride or docexatel. Therefore, this material is presented as a promising alternative to be used for administration and controlled release of drugs, for example, in cancer therapies.

**Figure 5:**
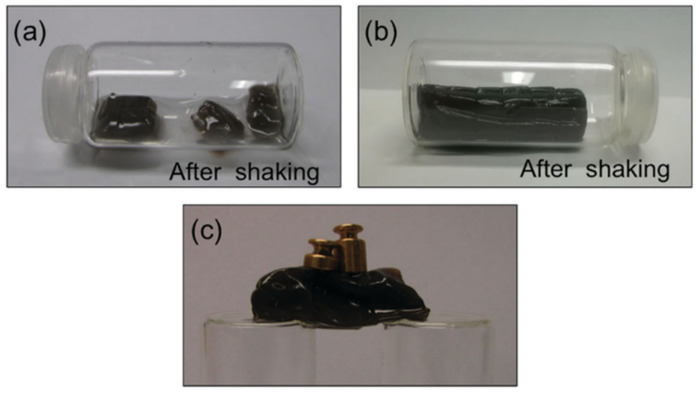
(**a**) Disruption of a hydrogel based on modified alginate with dopamine (DA) and (**b**) stable hydrogel based on catechol-polymerized alginate-DA. (**c**) Catechol-polymerizedalginate-DA hydrogel’s ability to bear a load 1.13 times greater than its own weight. Reprinted from J. Alegre-Requena et al. Regulatory parameters of self-healing alginate hydrogel networks prepared via mussel-inspired dynamic chemistry [104]. Published by The Royal Society of Chemistry, 2016.

Not only chitosan but also other polysaccharides have also been modified with catechol groups so as to obtain self-healable hydrogel by coordinative interactions. This is the case of modified alginate with dopamine (Alg-DA). A recent study compares the self-healing of the traditional ionic gel composed of unmodified alginate/Ca^2+^ and two gels formed in situ by coordinative interactions between modified Alg-DA and Fe (III) and polymerized-catechol Alg-DA and Fe (III) [104]. The results demonstrated that although all hydrogels were capable of self-healing, only dopamine-modified gels were capable of establishing bridges strong enough not to break under vigorous mechanical agitation (Figure 5). This behavior is due to the fact that the self-healing of dopamine-modified gels are able to bind strongly with iron (III) ions, thus giving the hydrogel more consistency after breaking.

### 3.3. In Situ Polymerization and Self-Healing Hydrogels

In situ hydrogels that are able to be self-repaired often do not show good mechanical stability and, in addition, they have poor self-repair ability, which strongly limits their application. Thus, as physical interactions typically used in the development of in situ hydrogels with self-healing due to their reversible nature promote the lack of mechanical stability, the interest in incorporating covalent bonds by in situ polymerization in physically crosslinked networks has increased. Most of the hydrogels based on in situ polymerization are based on a double crosslinking network. In fact, in situ polymerization generates a covalent network with improved mechanical stability. Since in most cases this network is not biodegradable, they frequently appear in combination with natural polysaccharides and form hydrogels based on reversible physical interactions that offer the possibility of self-repair. Related to this, usually this kind of material needs external stimuli such as temperature to complete self-healing process. This is the case, for example for double crosslinked hydrogels formed by in situ free radical polymerization and hydrogen bonds based on *N*-acryloyl glycinamide [105]. The hydrogels show good mechanical stability for the desired biomedical applications thanks to the covalent crosslinked and self-healing process being successfully achieved with the help of an external temperature, which favors the H-bonds between the residues of amino acids in the polymer. However, this hydrogel is unsuitable for biomedical applications because although it showed good cytocompatibility and mechanical stability (1400% elongation at break) the nature of the matrix does not present biodegradability.

**Figure 6:**
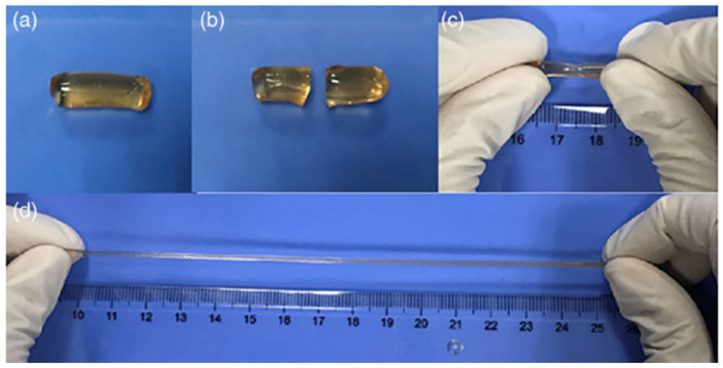
Self-healing experiments. (**a**) Original hydrogel. (**b**) Original hydrogel after being cut. (**c**) Self-healed hydrogel after healing for 2.5 h and (**d**) stretching of self-healed hydrogel. Reprinted from self-healing and conductivity of chitosan-based hydrogels formed by the migration of ferric ions [106]. Copyright © 2020 Wiley Periodicals, Inc.

**Figure 7:**
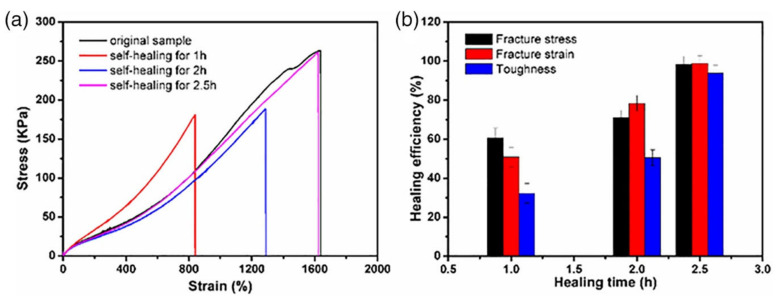
(**a**) Mechanical measurements of the self-healable hydrogel in different curing cycles. (**b**) Self-healing efficiency. Reprinted from self-healing and conductivity of chitosan-based hydrogels formed by the migration of ferric ions [106]. Copyright © 2020 Wiley Periodicals, Inc.

Nevertheless, multiple coordination bridges established by coordination metals are a promising way to add a second reversible network to covalently crosslink hydrogels, and thus, to gain high mechanical stability as well as an efficient self-repairing ability. Typically, chemically crosslinked hydrogels are obtained by in situ polymerization of poly (acrylic acid), polyacrylamide or their copolymers that are also coordinated with metallic cations like Fe^3+^ to add physical crosslinking points due to electrostatic interactions to the polymerized networks [106]. As aforementioned, the use of synthetic polymers for biomedical research is limited and, therefore, in situ hydrogels with self-healing properties based on polysaccharides are increasingly investigated. As aforementioned, chitosan is one of the most used biopolymers for the development of hydrogels based on polysaccharides. For example, double-crosslinked chitosan hydrogels were prepared by combining the in situ free-radical polymerization of acrylic acid monomer and reversible coordinative interactions between water soluble chitosan and Fe^3+^ ions that provide a hydrogel with good mechanical properties and ability to self-repair [106]. The results showed that in situ hydrogels based on chitosan had excellent efficiency for self-repair (98%) and were able to achieve total self-repair in just 2.5 h (Figure 6). Finally, the mechanical properties of the hydrogels after being fractured were compared with the original hydrogel and it was seen that after 2.5 h of self-healing, there were no changes in the mechanical properties, for example, the elongation at break could reach up to 1900% and its maximum tensile strength was about 280 kPa in both cases (Figure 7).

As aforementioned, an acid medium is often necessary to dissolve chitosan. However, its primary amino group allows easy functionalization. This is the case of quaternized chitosan which, in addition to possessing a high charge density due to a high degree of substitution, has very good solubility in water. Therefore, quaternized chitosan has been successfully used in the development of hybrid hydrogels based on a double crosslinking [107]. On the one hand, direct in situ polymerization of acrylic acid (AA) monomers within a concentrated solution of quaternized chitosan and, on the other hand, ionic crosslinking between the positive charges of the quaternized chitosan and the negative charges of poly (acrylic acid) (PAA). The results revealed that these hydrogels, in addition to presenting very good mechanical stability due to the strong electrostatic interactions between the polysaccharide and the PAA (maximum stress 16.1 MPa), also had the capacity to recover their initial shape after rupture in just 1 min after having them immersed in water. The self-repairing process was primarily driven by electrostatic interactions between quaternized chitosan and PAA.

Another type of hybrid hydrogels that could be included in this classification is based on alginate and polyacrylamide [108]. Thanks to alginate, a reversible non-covalent network is achieved based on ionic interactions between alginate and divalent ions (Ca^2+^) through which self-repair is achieved. On the other hand, to provide the gel with good stability and mechanical resistance, a double covalent network is added, based on an in situ photopolymerization between the polyacryamide and the monomer *N*,*N*-methylenebisacrylamide. The results of the mechanical tests revealed that during elongation the covalent bonds remain stable (they can stretch 20 times their initial length) and it is the ionic bonds that dissipate energy by breaking. These hybrid hydrogels reach up to 9000 J m^−2^ when other synthetic hydrogels have hardly reached 1000 J m^−2^ [109,110,111]. Thanks to the ionic bonds, the gel was able to recover its initial shape by up to 74% after one charge-discharge cycle.

### 3.4. In Situ Hydrogels Formed via Dynamic Covalent Bonds with Self-Healing

**Figure 8:**
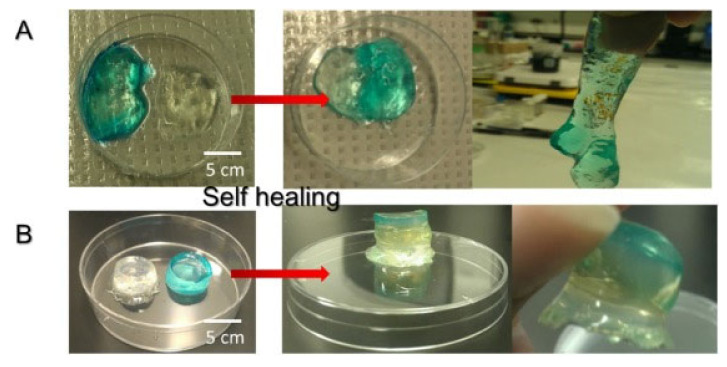
Scheme of self-healing process of sodium alginate/cellulose nanocrystal (CNC)-based (**A**) two component hydrogel and (**B**) three-component hydrogel. Reprinted from self-healing stimuli-responsive cellulose nanocrystal hydrogels [43]. Copyright (2019) with permission from Elsevier.

Click chemistry is one of the most exploited strategies for the development of smart in situ hydrogels, specifically for the development of self-healing hydrogels. Different polysaccharide-based hydrogels were obtained in situ by click chemistry with capacity to self-repair. For instance, alginate has been successfully used so as to obtain scaffolds with enhanced biodegradability and biocompatibility [43]. As has been commented above, physically crosslinked hydrogels usually do not have good mechanical stability so, in order to obtain more consistent hydrogel, some researchers added cellulose nanocrystals (CNCs). Initially, polysaccharides and the CNCs, to get the aldehyde groups necessary for the Schiff reaction, were oxidized and subsequently, vinyl monomers modified with amine groups were introduced to the CNC surface and backbone of the alginate using the Schiff-base reaction. Hydrogels with a homogeneous chemical structure were formed in a few minutes with self-heal ability in 3 h at room temperature thanks to the hydrogen bonding and chain entanglements (Figure 8).

**Figure 9:**
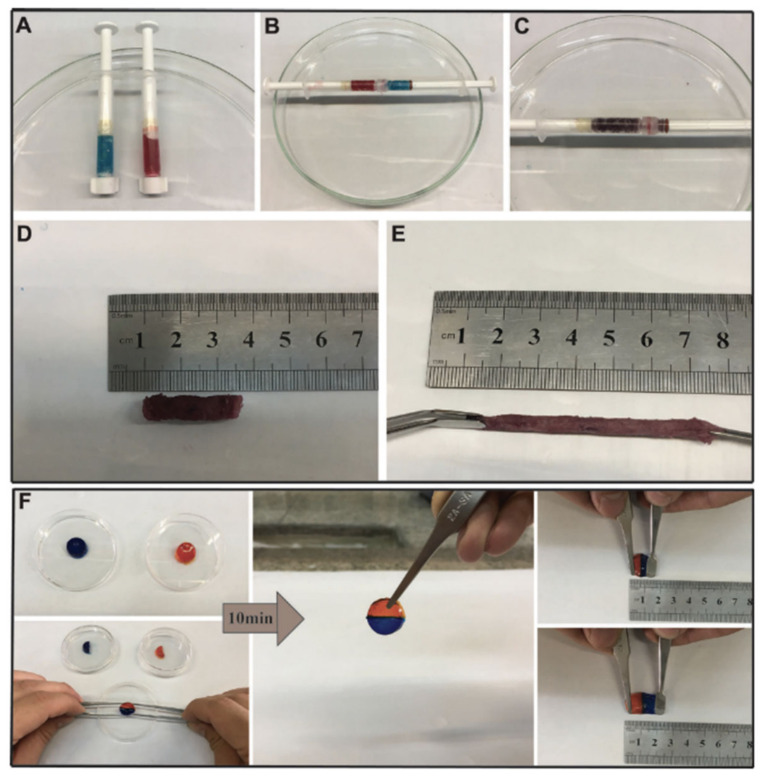
Self-healing process. (**A**–**E**) Self-healing behavior through a syringe, mixing the two halves of the hydrogel. (**F**) Visual self-healing connecting two halves of a disk hydrogel. Reprinted from M. Vahedi et al. Self-Healing, Injectable Gelatin Hydrogels Cross-Linked by Dynamic Schiff Base Linkages Support Cell Adhesion and Sustained Release of Antibacterial Drugs [112]. Copyright © (2018) with permission of Wiley-VCH.

Other polysaccharides, such as gelatin, have also been employed in the development of in situ self-healable hydrogels. Vahedi et al. [112] developed a new gelatin-based in situ hydrogel prepared by Schiff-base reaction. Gelatin is a natural, biocompatible and biodegradable polysaccharide that presents amino groups in its backbone which are suitable for reacting with poly(ethylene glycol) di-benzaldehyde without the need for borax or any other type of chemical agent. The hydrogels are created in no more than 20 s and show high capacity to be injected, as well as quick self-repair (10 min) without the need of any external factor (Figure 9) thanks to the dynamic imine bonds. This rapid self-healing and ability to be injected confers to the hydrogel the opportunity to be extruded through a syringe which, as shown in Figure 9, the mixture of half of the hydrogels merged perfectly due to the combination of both colors.

**Figure 10:**
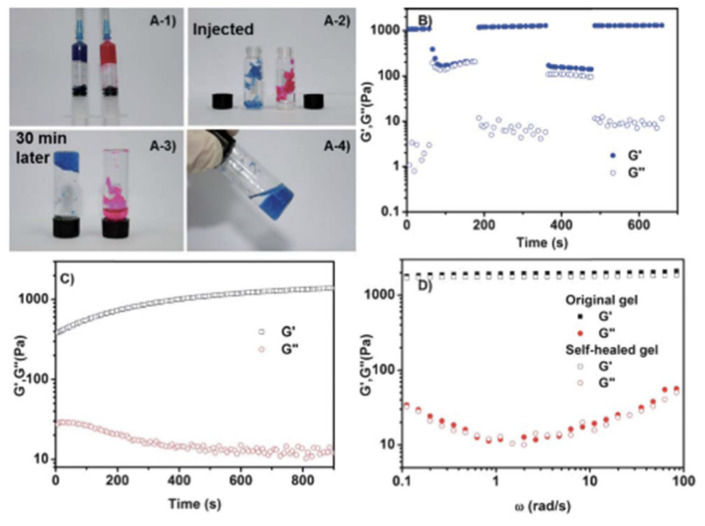
(**A**) Self-healing after injection. (**B**–**D**) Rheological characterization of (**B**) Chitosan/Poly/ethylene glycol hydrogel deformation and recovery. G′ (filled symbols) and G″ (empty symbols). (**C**) G′ and G″ with time during the self-healing process. (**D**) Comparison of original and self-healed hydrogels. Reproduced by permission of The Royal Society of Chemistry from B. Yang et al. Facilely prepared inexpensive and biocompatible self-healing hydrogel: a new injectable cell therapy carrier [56].

Chitosan has also been selected on numerous occasions for the formation of dynamic bonds by the Schiff-base reaction. Glycol chitosan (GCH), due to its improved solubility compared to traditional chitosan, has been used together with telechelic difunctional poly(ethylene glycol) (DF-PEG) to develop in situ hydrogels capable of self-healing [56]. Thanks to the -NH_2_ groups of the polysaccharide chain and to the benzaldehyde groups of DF-PEG, dynamic Schiff-base covalent bonds were satisfactorily established, resulting in the gelation of the material in just 1 min. As discussed previously, due to the quasi-covalent nature of Schiff-base mediated bonds, hydrogels were expected to self-repair. That is why to verify it, the traditional study of cutting the hydrogel in two halves was left aside and studying the self-healing process after being injected was chosen, that is, it was expected that when adding pressure and extruding the material, the links break and when the pressure stops the links form again. To do this, they stained the hydrogel (GCH-DF-PEG) with blue and put it in a syringe. Meanwhile, in another syringe they put a traditional gelatin gel stained with rhodamine B, thus acquiring the pink color seen in Figure 10. As can be seen in the aforementioned figure, the evidence of the self-repair process is clear. After injection, only the blue gel (GCH-DF-PEG) was able to regenerate again. That process lasted around 30 min and without the help of any external stimulation thanks to the reversible Schiff-base bonds.

**Figure 11:**
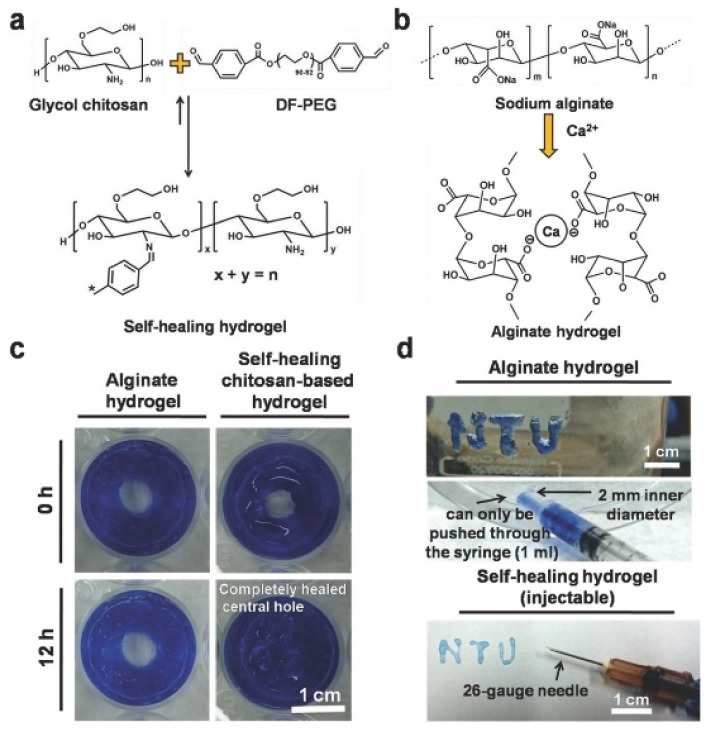
Alginate-based hydrogel’s completed recovery. (**A**) Benzaldehydes based hydrogels to form self-healable hydrogels. (**B**) Alginate based hydrogels crosslinked with calcium chloride (without self-healing property). (**C**) Gross appearance of alginate hydrogels and self healing hydrogel. (**D**) Self-healing hydrogel is able to be extruded by 26 gauge needle. Reproduced from An injectable, self-healing hydrogel to repair the central nervous system [113].

Other studies have also relied on GCH-DF-PEG hydrogels for biomedical applications so as to obtain in situ self-healing hydrogels [113]. In the case of Figure 11, the self-healing process is studied in a different way from that shown in Figure 10. In this case, the GCH-DF-PEG gels were able to regenerate a hole in the center of the material while the alginate hydrogels used in this case as a control could not regenerate. Once again, it is clear that the Schiff-base bonds are pseudo-covalent which makes them capable of re-establishing after fracture. More recently, hyaluronic acid has been included to GCH-DF-PEG hydrogels leading to semi-interpenetrating polymer network with improved injectability and differentiation of in vitro loaded neural stem cells [114].

**Figure 12:**
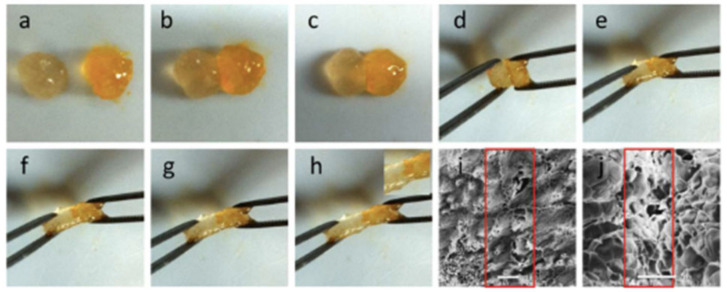
(**a**–**h**) Self-healing process of *N*-succinyl chitosan and chondroitin sulfate multiple aldehyde based injectable hydrogel. SEM images of healed hydrogels (**i**): surface, ×70, scale bar = 200 μm; (**j**): internal, ×500, scale bar = 50 μm. Reprinted with permission from Injectable and Self-Healing Carbohydrate-Based Hydrogel for Cell Encapsulation [57]. Copyright © (2015), American Chemical Society.

**Figure 13:**
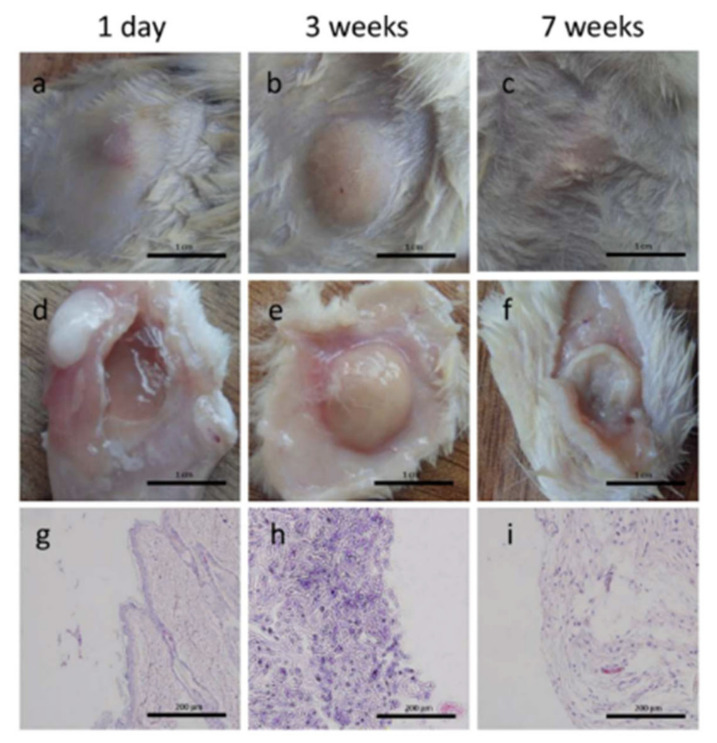
Biodegradation process of subcutaneously injected hydrogel based on *N*-succinyl chitosan and chondroitin sulfate multiple aldehyde. Scale bar of 1 cm for images (**a**–**f**) and 1 μm for images (**g**–**i**). Reprinted with permission from Injectable and Self-Healing Carbohydrate-Based Hydrogel for Cell Encapsulation [57]. Copyright © (2015), American Chemical Society.

Another derivative of chitosan, *N*-succinyl chitosan (SC), has also been used in the development of in situ hydrogels capable of self-regeneration in combination with chondroitin sulfate multiple aldehyde. Schiff-base bonds between aldehyde groups of chondroitin sulphate and amine group of chitosan caused the hydrogel to form in time ranging from 34 to 41 s depending on the molar ratios between the polysaccharide and the chondroitin sulfate. As in the previous cases, the self-healing process took place satisfactorily in just 2 h without the use of any external stimulus, and the results can be seen in Figure 12. Thanks to the cytotoxicity tests which show favorable results, this material, unlike the previous study, was tested in vivo on mice. Thanks to the fact that they are injectable, they could easily inject subcutaneously into the mice and none showed signs of local irritation. As time passes, the size of the hydrogel in the mouse’s skin decreased, making it clear that the biodegradation process was taking place (Figure 13).

**Figure 14:**
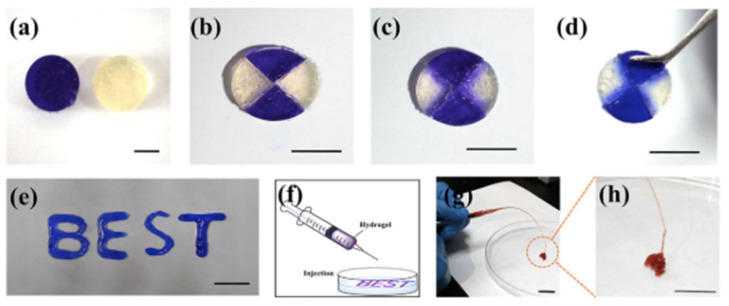
Self-healing process after dibenzaldehyde-terminated poly(ethylene glycol (PEG-DA)-based hydrogels were cut and joined. (**a**) Two disk-shaped original hydrogels, (**b**) Original hydrogels were cut in 4 pieces and put back together. (**c**,**d**) The completed healing of two original hydrogels. (**e**) Self-healed hydrogel extruded by a needle without clogging. (**f**–**h**) Illustration of the injection process of the hydrogels. Reprinted with permission from J. Qu et al. [36]. pH-responsive self-healing injectable hydrogel based on *N*-carboxyethyl chitosan for hepatocellular carcinoma therapy. Copyright © (2017), Acta Biomaterialia Inc. Published by Elsevier. All rights reserved.

As indicated before, in situ self-healing hydrogels with pH-responsiveness present as promising materials for bioapplications, in particular for those related with controlled release of drugs. Chitosan-derived *N*-carboxymetil chitosan was used combined with dibenzaldehyde-terminated poly(ethylene glycol) (PEG-DA) for the research of hepatocellular carcinoma [36]. The biocompatible hydrogel was synthesized thanks to dynamic covalent Schiff-base bonds between primary amine groups of chitosan and benzaldehyde groups of PEG-DA. The self-healing process was studied macroscopically and rheologically. As shown in Figure 14, the different color parts come together perfectly and there are even certain areas that acquire a bluish color, indicating that there is indeed molecular movement between the different parts of the hydrogel. This movement is given thanks to Schiff-base pseudo-covalent bonds that, as has been shown in previous studies, allow the hydrogel to self-repair. The hydrolytic degradation given due to pH sensitivity is explained by chitosan. As previously reported, the microenvironments in the tumor areas acquire a certain acidic pH that, considering the pKa of the chitosan is more or less 6.5, it would be protonated, positively charged. Therefore, the Schiff-base reaction between the primary amine group of chitosan and the aldehyde group of PEG-DA weakens and decomposition of the hydrogel takes place.

**Figure 15:**
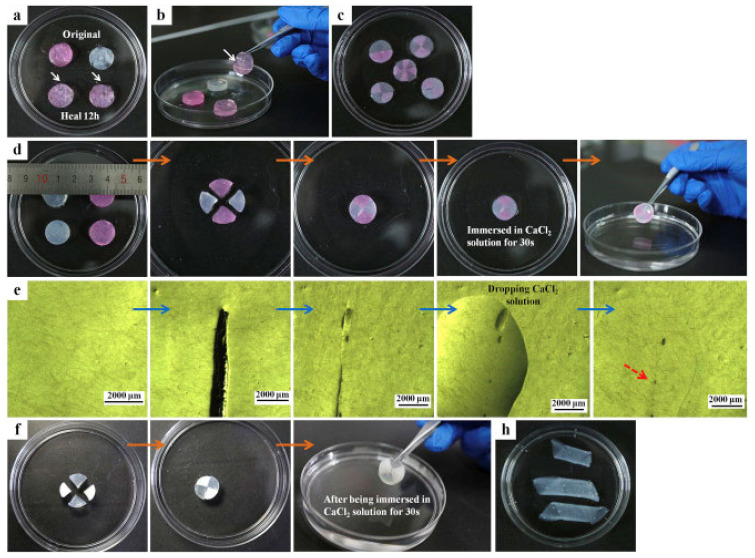
Photographs of self-healing process. (**a**–**c**) Healing process after 12 h and with different amount of pieces. (**d**) Optical microscopy images, (**e**) Rapid ionic self-healing process. (**f**) Self healing process of freeze-dried scaffold, (**h**) Tubular hollow structure of hydrogels. Reprinted with permission from B. Ye et al. [115]. An in situ formable and fibrils-reinforced polysaccharide composite hydrogel by self-crosslinking with dual healing ability. Copyright © 2020, Elsevier Ltd. All rights reserved.

Despite Schiff-base reaction is the most employed click reaction in the manufacturing of in situ hydrogels, Diels-Alder or Michael additions have been also reported for the synthesis of in situ and self-healable hydrogels. In the work by Ye et al. [112], a novel in situ polysaccharide-based hydrogel with maleitated chitosan and thiol derivatized sodium alginate was developed via Michael addition between vinyl groups and thiolated anions and ionic interactions between thiol-derivatized sodium alginate and Ca^2+^ [112]. This dual-crosslinked hydrogel can self-heal cracks due to disulfide exchange in no more than 12 h at physiological temperature without any external stimuli or chemical factors. Furthermore, the authors proved that the self-healing process could be favored by adding calcium ions, which accelerated the process until the cracks disappeared in 1 min (Figure 15) since the crosslinking takes place instantaneously. On the other hand, a dual crosslinked polysaccharide-based in situ forming biodegradable hydrogel was developed by substitution of the carboxylic groups of alginate with furan, which leads in the ability of the polysaccharide to react by Diels-Alder click reaction with poly(ethylene glycol) modified with maleimide end groups as a crosslinker. The results showed that the hydrogel could recover its original shape at incubating overnight at 37 °C and it is able to maintain its mechanical stability (70% of recovery) slightly the same as the original hydrogel thanks to the ionic interactions between furan-modified alginate and calcium divalent cations and dynamic covalent bonds between maleimide and furan-modified alginate.

Finally, it is worth noting that although the dynamic covalent bonds shown above are undoubtedly the most widely used in the development of in situ hydrogels capable of healing, the boronic ester bonds that occur when reacting groups of phenyl-boronic acids (PBAs) and 1,2 and 1,3 diols are also an attractive option towards the development of self-healing biomaterials for biomedical applications. This is the case, for example, of a hydrogel based on different derivatives of the polysaccharide hyaluronic acid. Firstly, hyaluronic acid was modified with maltose and with PBA groups. PBA has the ability to react cis-diol units and therefore it was expected that it could modify the polysaccharide easily. However, this did not allow the PBA groups to successfully complex as it happened with other neutral sugars such as glucose and, therefore, different researchers have chosen to add terminal glucose to each repetitive unit of the polysaccharide chain promoting the modification of hyaluronic acid. Once the polysaccharide had been modified with the different groups, the corresponding hydrogel formed in aqueous solution under temperature and physiological pH almost instantaneously. Furthermore, it should be noted that it is commonly accepted that the ideal pH for boronate esterifications is around the pKa of the species that contains these groups. However, in the case reported above it was successfully achieved at neutral pH because hyaluronic acid, being a polyanion, decreased the pKa of the boronic acid units due to the interactions that were established between the carboxylic groups of hyaluronic acid and the PBA. To test the reversibility of these dynamic covalent bonds, the self-healing process of the hydrogels was studied. The gel showed almost immediate self-repair after stressing it out. Furthermore, after five cycles of cut-heal the gel remained stable showing that it did not lose its characteristic mechanical stability, since the elastic modulus values remain intact (~1000 Pa).

Other studies have also corroborated the ability of these pseudo-covalent bonds to develop self-healing hydrogels. For example, alginate has also been used successfully in the synthesis of these type of biomaterials. As in the previous study and taking advantage of the fact that alginate has alcohol groups along its chain, it is modified with boronic acid groups to promote the conjugation between the carboxyl groups of the polysaccharide and the anime groups of the boronic for the creation of dynamic boronic ester bonds. The hydrogel formed in just a few minutes thanks to the links established in the cis-diols of the alginate chain with the modified BA groups in the polysaccharide. Thanks to the reversibility of the dynamic bonds established between the BA and alginate diols, it was possible to self-repair without the help of any external stimulus in just 5 min. On the other hand, it is typical that covalently cross-linked three-dimensional networks suffer fractures at high stresses due to their rigidity. However, this hydrogel could be stretched 23 times in the longitudinal direction (23 cm) and left no evidence of fracture. This behavior, however, was not seen in the traditional alginate hydrogel (alginate/Ca^2+^).

## 4. Tissue Engineering Applications

The main goal of tissue engineering is the restoration of degenerated or damaged tissues, being the materials used capable to mimic, until certain degree, the native tissue in order to restore its functionally and structure [116]. Commonly, the materials used in tissue engineering could be divided into three groups: scaffolds, cells and growth factors. Among other possible materials, hydrogels, as has been described in this review, present excellent properties to be used for scaffold construction.

### 4.1. Bone Tissue

The populations of many developed countries are getting significantly old. Elderly people present frequently health issues related to bone defects, being in many cases one of the causes of disability in this group of population [117]. Bone tissue engineering could be considered as a promising approach for bone repairing. In this context, self-healing scaffolds, built with injectable hydrogels, are considered as emerging materials. These materials could be adapted to the load conditions in the native bone and facilitate the growth of the new tissue.

Zhang et al. [118] reported the fabrication of in situ forming nanocomposite hydrogel based on coordinative interactions between magnesium cations (Mg^2+^) and bisphosphonate (BP). Hyaluronic acid modified with BP and acrylated-BP (Ac-BP) was used to promote the coordination with Mg cations forming dynamic interactions that provide self-healing capability. In addition, the spontaneous coordination of Ac-BP with Mg^2+^ induces the formation of nanoparticles capable of stabilizing these in situ forming hydrogels due to their multivalent crosslinkering capability. These hydrogels present enhanced mechanical properties. Although, these properties could be improved by the photopolymerization of the acrylamide groups present in the nanoparticle, increasing the stiffness. However, the biological test carried out by encapsulating human mesenchymal stem cells (hMSCs) on the hydrogels non-photo crosslinked and crosslinked indicated that the additional UV-crosslinking inhibits cell spreading and osteogenesis.

**Figure 16:**
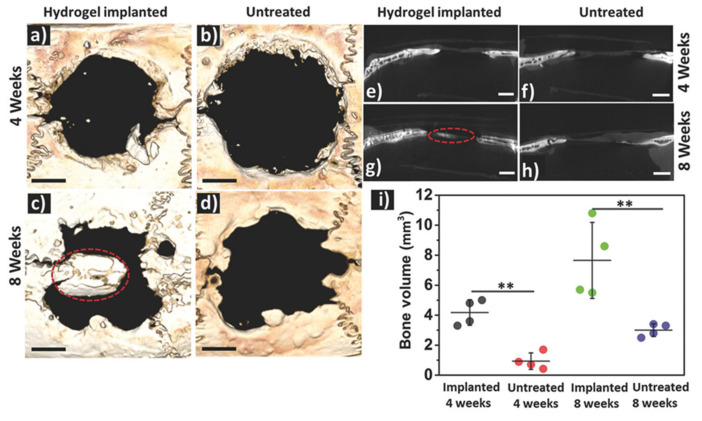
Bone formation in a rat cranial defect in untreated and after implantation of SF-based hydrogel: (**a**–**d**) 3D reconstruction views of the studied defect, (**e**–**h**) sagittal cross-section of the bone and (**i**) formed bone volume per defect (** *p* < 0.01). Reproduced by permission from Shi et al. [119]. Copyright 2017, Wiley-VCH.

Similarly, Shi et al. [119] reported the synthesis of silk fibroin based injectable hydrogels for bone regeneration. These hydrogels present a self-healing capability and shear-thinning characteristic due to their dynamic metal-ligand self-assembly, that is, dynamic metal-bisphosphonate coordination bonds. In this work, previously synthetized microfibers of silk fibroin (mSF) (length 200 µm and diameter 10 µm) were coated with calcium phosphate (CaP) under mild biomineralization. These biomineralized microfibers (CaP@mSF), which are chelated by bisphosphonate ligands of the binder, are capable of forming reversible crosslinkages, the drive force for the self-healing capability of the hydrogels. In order to obtain these reversible bonds by chelation with the calcium present, CaP-mSF, the hyaluronic acid, used as polysaccharide binder, was modified for the bisphosphonate groups. In addition, this hydrogel presents some mechanical weakness, so this property was improved by photocrosslinking of the HA binder with UV radiation, increasing the storage modulus up to ≈1500%. In this step, HA was functionalized with BP and acrylamide (Am-HA-BP), being this functional group capable of photopolymerizing. After the photocrosslinking process, this SF-based hydrogel presentde a robust double network that could be considered as advantageous to bone regeneration applications. The study demonstrated that these hydrogels present good cell viability and they are able to support stem cell growth. Finally, the in vivo test demonstrated the efficiency of the SF-based hydrogel for bone regeneration. The hydrogels were implanted into a cranial defect of a rat, and the evaluation of the implant was carried out after 4 and 8 weeks. As can be observed in Figure 16, the bone regeneration presents a significant acceleration induced by the hydrogel injection with a bone formation rate 220% faster than in the reference case.

**Figure 17:**
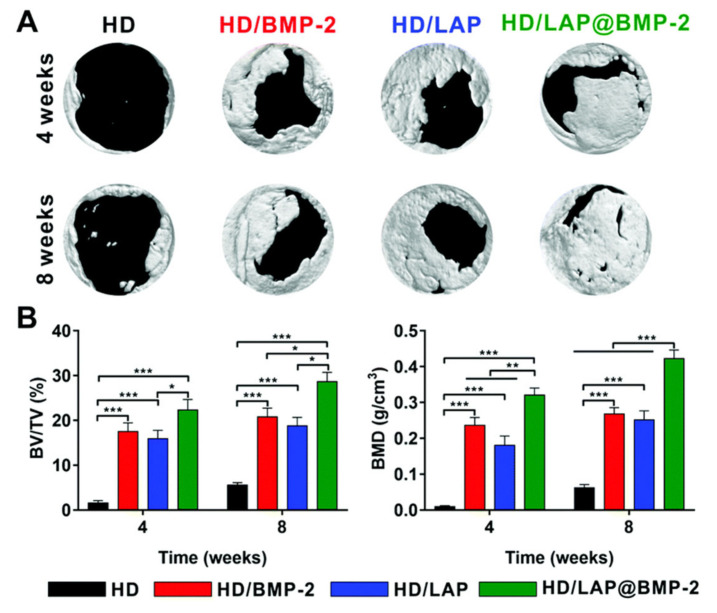
(**A**) In vivo study of the evaluation of calvarial bone defect regeneration at 4 and 8 weeks after the implantation and (**B**) analysis of bone volume/total volume (BV/TV) and bone mineral density (BMD) (* *p* < 0.05, ** *p* < 0.01 and *** *p* < 0.001). Reproduced with permission from [120] (Copyright RSC 2020).

Another example of self-healing hydrogel nanocomposite for bone tissue regeneration based on hyaluronic acid and dextran has been reported [120]. This hydrogel could be in situ formed through a spontaneous Schiff-base reaction. Hydrogels are loaded with LAPONITE^®^ (LAP) nanoplatelets that form hydrogen bonds and accelerate gel formation, improving the mechanical properties of the hydrogels. In addition, the hydrogels incorporate bone morphogenetic protein 2. This protein could be complexed with the LAP forming LAP@BMP-2 complexes and preserving the bioactivity of this protein. In addition, the in vitro and in vivo tests indicated that this complex improves proliferation activity and osteogenesis. In Figure 17, the bone regeneration obtained for the in vivo test could be observed after 8 weeks. In this study, the hydrogels with LAP@BMP-2 complexes are compared to LAP and BMP-2 hydrogels.

Gelatin derived self-healable hydrogels have also been explored as osteoinductive scaffolds. Hydrogels prepared by Shiff-base reaction between amylopectin multiple aldehyde and gelatin, apart from showing good injectability and self-healing ability, induced osteogenic differentiation of human bone marrow stromal cells (hBMSCs) in absence of osteoinductive supplements [121].

### 4.2. Cartilage

Trauma, arthritis or injuries derived from sport practice often evolve on articular cartilage damages, making hydrogels very interesting materials for the treatment of these damages due to their capability to promote its regeneration. Hou et al. [122] developed a biocompatible injectable hydrogel based on dextran functionalized with Ureido-pyrimidinone (Upy) that enables the formation of strong reversible hydrogen bonds. The quadruple hydrogen bond associated with the Upy provides self-healing and shear-thinning properties to this hydrogel. The authors mixed together two hydrogels, one with chondrocytes for cartilage formation encapsulated in the hydrogel and another with bone morphogenetic protein 2 (BMP-2) for bone regeneration encapsulated in order to generate an artificial cartilage-bone interface. After 8 weeks of implantation, the results demonstrated the suitability of this hydrogel for supporting the capability of the new hydrogel in supporting the growth of both bone and cartilage tissues, that is, the cartilage-bone tissue complex was successfully regenerated.

Similarly, Yu et al. [123] reported that double-crosslinked hydrogels were capable of integrating themselves on host cartilage. The proposed system was based on Diels-Alder click reaction and acylhydrazone bond as two main interactions for a double network hydrogel. Each interaction provides one function to the hydrogel. The Diels-Alder reaction is the one responsible for the mechanical strength, whereas the dynamic nature of acylhydrazone bond induces the self-healing property. In addition, the Schiff-base reaction between the aldehyde groups and amide groups present in the surrounding tissues eases the adhesion and the integration of the hydrogels with the cartilage tissues. These hydrogels, with double network in their structure, present a higher compressive modulus compared to the formulations with a single network.

### 4.3. Muscle

Guo and co-workers [124] fabricated electroactive injectable hydrogels with self-healing capability for tissue engineering and for skeletal muscle regeneration. These chitosan-based hydrogels present electroactive properties due to the dextran-graft-aniline tetramer-graft-4-formylbenzoic acid. The in situ forming and self-healing capacity is obtained due to a dynamic Schiff-base bonds formed between the formylbenzoic acid and amine groups of the chitosan. These formulations present a good in vivo injectability. The culture of L929 fibroblast indicated that hydrogels present a good biocompatibility. The C2C12 myoblasts cells encapsulated and the culture in the hydrogels were released from them following a lineal profile. The obtained results indicated that hydrogels promoted the skeletal muscle reparation.

### 4.4. Skin

The skin is the organ in which the first defense line of the body lies, so it often presents damages due to the daily activities. However, some patients present skin healing problems or chronic wounds. Hydrogels could provide an adequate environment for improving the wound-healing process [125,126].

**Figure 18:**
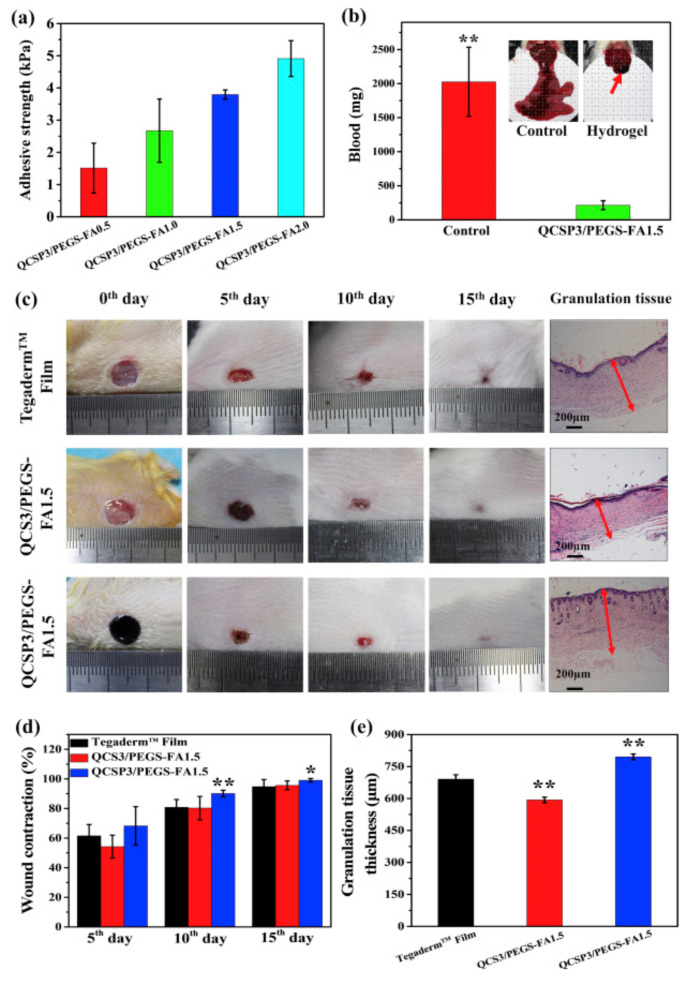
(**a**) Adhesion test of the hydrogels, (**b**) hemostatic study, (**c**) wound-healing capability of the hydrogels and (**d**) wound contraction once the hydrogels were implanted and (**e**) granulation tissue thickness (* *p* < 0.05, ** *p* < 0.01). Reproduced with permission from [127]. Copyright 2017 Elsevier.

Zhao et al. [127] developed an injectable and conductive hydrogel based on quaternized chitosan-*g*-polyaniline (QCSP) and poly(ethylene glycol)-*co*-poly(glycerol sebacate) functionalized with benzaldehyde (PEGS-FA). These hydrogels present good adhesion and self-healing properties, in addition to antibacterial and anti-oxidant properties to make them highly suitable for wound-repairing applications. It is important to notice that one of the tested formulations, 1.5% wt of PEGS-FA, present an excellent blood clotting capability. Additionally, authors in vivo studied the wound dressing performance on a skin-defect in a mouse model for 15 days, resulting in a higher expression of wound-healing markers (EGF, TGF-β, and VEGF). This result was significantly better than the result obtained for a commercial wound dressing (Tegaderm). Figure 18 summarized some of the main results obtained in this work.

Rapid and robust self-healing properties were demonstrated by the hydrogels obtained by Schiff-base reactions between aldehyde groups of modified methyl cellulose and the amine groups of PEG-grafted chitosan. The gels were explored as scaffolds for the loading of exosomes and this combination led to a synergistic improvement of their wound healing ability [128].

### 4.5. Cardiac

Heart attack or acute myocardial infarction is one of the important mortality causes that occurs when the blood flow does not arrive to the heart and causes tissue damages. Among the possible approaches described for the prevention of a second infarction, the application of cell therapy with injectable hydrogels could be considered a highly suitable alternative [129,130]. Dong et al. [131] developed a conductive injectable based on chitosan and polyethylene glycol hydrogel for cell delivery. The hydrogel was formed with chitosan-graft-aniline tetramer (CS-AT) and dibenzaldehyde-terminated poly(ethylene glycol) (PEG-DA). Once the two components were mixed at physiological temperature, a self-healable hydrogel was created, presenting a good injectability. This hydrogel presents a conductivity close to 10^−3^ S·cm^−1^ and good tissue adhesion tested with a porcine skin model. In addition, the biocompatibility test with C2C12 myoblasts was good, presenting an adequate proliferation, and being the viability of the C2C12 not reduced after the injection. Finally, an in vivo test was carried out with two different cells encapsulated in the hydrogels C2C12 myoblasts and H9c2 cardiac cells. The test showed a good release profile and good biodegradation of the injectable hydrogels. The results demonstrated that these hydrogels could be considered as very promising cell carriers for cardiac repair.

Bastings et al. [132] fabricated an in situ forming hydrogel capable of being injected through a catheter based in polyethylene glycol derivatives functionalized with UPy. The hydrogen bonds formed by the UPy moieties gave the self-healing capability to the hydrogel. These interactions are pH sensitive, being the hydrogel fluid and injectable at basic pH and solid hydrogel at neutral pH. In addition, these hydrogels were loaded with growth factors (HGF and IGF-1) and injected in a porcine myocardial infarction mode. The results suggested that the hydrogels could reduce the size of the infarct scar and active resident regenerative cells, promoting cardiac tissue regeneration.

Finally, Table 1 summarized the composition, self-healing mechanisms and applications of described hydrogels.

## 5. Conclusions

Biocompatible and biodegradable hydrogels that are able to be self-repaired from mechanical damages are of great interest to be applied as biomedical implantable systems due to this ability, prolonging their integrity and lifetime. When, together with this property, injectability is achieved, the applicability of the obtained materials increases substantially, since invasive implantation is avoided, while personalized application is possible. Hence, the interest in durable, self-healable and injectable hydrogels based on natural polysaccharides has led to a fructiferous research area with promising results for tissue engineering applications. For this, a wide variety of dynamic interactions, both physical and chemicals, has been identified in the last decade as driving forces to provide both in situ gelation and self-healing properties. The compromise between rapid self-healing behaviors and adequate mechanical properties makes the design and development of these types of hydrogels a challenging issue nowadays. Accordingly, the future perspectives of these specific hydrogels point to the searching of new associations of materials (networks, polymers, nanoparticles, fibers, among others) to overcome current challenges and develop new systems to be applied as scaffolds and implants for personalized biomedicine applications. Fortunately, the combination of different crosslinking and dynamic forces, as well as different polysaccharides with both natural and synthetic polymers, has made possible the development of hydrogels with the mentioned attributes in a feasible way. Furthermore, these specific combinations have demonstrated additional valuable properties such as the ability to respond to environmental changes, which is of great importance for tissue engineering applications.

## Figures and Tables

**Table 1:** Summary of the composition, self-healing mechanisms and applications of described hydrogels.

Hydrogel	In Situ Gelling Mechanism	Self-Healing Mechanism	Applications	References
Alginate grafted onto β-CD and Pluronic^®^ F108	Hydrophobic interactions	Host-guest interaction	Drug delivery and cell transplantantion	[97]
Hydrophobically modified chitosan with thermal-responsive vesicle composed of 5 mS and DTAB	Hydrophobic interactions	Host-guest interaction	Scaffold for tissue engineering	[98]
Polyethylene glycol derivated functionalized with/ureido-pyrimidinone	Hydrophobic interactions	Hydrophobic interactions	Cardiac tissue regeneration	[132]
2-Hydroxypropyltrimethylammonium chitosan chloride/Alginate polycomplex	Electrostatic interactions	Ionically induced mechanism	Surface coating and 3D bioprinting	[102]
Chitosan/Hyaluronic acid polycomplex	Electrostatic interactions	Ionically induced mechanism	Functional biomedical supports and coatings	[51]
Polyacrylic acid (PAA)—chitosan copolymeric hydrogels crosslinked with guanidine	Electrostatic interactions	Ionically induced mechanism	Drug delivery and tissue engineering applications	[103]
Chitosan/poly(acrylamide-co-acrylic acid) in presence of Fe^3+^	Electrostatic interactions	Ionically induced mechanism	Drug delivery and tissue engineering applications	[90]
Dextran functionalized with Ureido-pyrimidinone	Hydrogen bonds	Hydrogen bonds	Encapsulation of chondrocytes for cartilage tissue engineering	[122]
Chitosan/Catechol assembled through Fe^3+^	Metal-ligand interactions	Ionically induced mechanism	Localized delivery of chemotherapeutic drugs	[133]
Alginate/Catechol assembled through Fe^3+^	Metal-ligand coordinative interactions	Ionically induced mechanism	Drug delivery and tissue differentiation	[104]
*N*-carboxyethyl chitosan/Polyacrylic acid coordinated with Fe^3+^	Metal-ligand coordinative interactions	Ionically induced mechanism	Drug delivery and cartilage tissue differentiation	[106]
Hyaluronic acid modified with bisphosphonate and acrylated bisphosphonate and Mg^2+^	Metal-ligand coordinative interactions	Metal-ligand coordinative interactions	Bone tissue engineering	[118]
Hyaluronic acid hydrogel with silk fibroin nanofibers coated with calcium phosphate	Dynamic metal-bisphosphonate coordination bonds	Dynamic metal-bisphosphonate coordination bonds	Bone tissue engineering	[119]
Quaternized chitosan/Polyacrylic acid	In situ polymerization of monomer and electrostatic interactions	Ionically induced mechanism	Load-bearing artificial soft tissues.	[107]
Alginate/Ca^2+^/Polyacrylamide	In situ polymerization and electrostatic interactions	Ionically induced mechanism	3D cell culture	[108]
Gelatin/polyethylene glycol dibenzaldehyde	Dynamic imine bonds	Dynamic imine bonds	Drug delivery systems, as tissue regeneration scaffolds and also as wound dressing for skin injuries	[112]
Sodium alginate dialdehyde/Carboxymethyl chitosan	Dynamic Schiff-base covalent bonds	Hydrogen bonds and dynamic Schiff-base covalent bonds	Corneal epithelial reconstruction based on tissue engineering	[43]
Glycol chitosan/telechelic difunctional poly(ethylene glycol)	Dynamic Schiff-base covalent bonds	Dynamic Schiff-base covalent bonds	3D cell culture and injection cell therapy	[56]
*N*-carboxyethyl chitosan/dibenzaldehyde-terminated poly(ethylene glycol)	Dynamic Schiff-base covalent bonds	Dynamic Schiff-base covalent bonds	Anticancer drug delivery for hepatocellular cancer therapy	[36]
Hyaluronic acid/Dextran/LAPONITE^®^	Dynamic Schiff-base covalent bonds and hydrogen bonds	Dynamic Schiff-base covalent bonds and hydrogen bonds	Bone tissue engineering	[120]
Chitosan/dextran-graft-aniline tetramer-graft-4-formylbenzoic acid	Dynamic Schiff-base covalent bonds	Dynamic Schiff-base covalent bonds	Encapsulation of C2C12 myoblasts for muscle tissue engineering	[124]
Quaternized chitosan-g-polyaniline and poly(ethylene glycol)-*co*-poly(glycerol sebacate) funtionalized with benzaldehyde	Dynamic Schiff-base covalent bonds	Dynamic Schiff-base covalent bonds	Wound repair	[127]
Chitosan-*graft*-aniline tetramer and dibenzaldehyde-terminated poly(ethylene glycol)	Dynamic Schiff-base covalent bonds	Dynamic Schiff-base covalent bonds	Regeneration of cardiac tissue for myocardial infarction	[131]
Hyaluronic acid/furan/adipic dihydrazide	Diels alder click reaction and dynamic acylhydrazone bonds	Dynamic acylhydrazone bond	Cartilage tissue engineering	[123]
Maleilated chitosan/Thiol derivatized sodium alginate	Michael addition reaction and ionic interactions	Dynamic disulfide exchange	3D cell culture	[115]
Polyethylene glycol derivatives functionalized with/ureido-pyrimidinone	Hydrophobic interactions	Hydrophobic interactions	Cardiac tissue regeneration	[132]
